# Gβγ translocation to the Golgi apparatus activates ARF1 to spatiotemporally regulate G protein–coupled receptor signaling to MAPK

**DOI:** 10.1016/j.jbc.2021.100805

**Published:** 2021-05-19

**Authors:** Mostafa Khater, Christian N. Bryant, Guangyu Wu

**Affiliations:** Department of Pharmacology and Toxicology, Medical College of Georgia, Augusta University, Augusta, Georgia, USA

**Keywords:** G protein–coupled receptor, CXCR4, G protein, Gβγ, translocation, ARF1, Golgi, PI3Kγ, signaling, MAPK, ARF, ADP-ribosylation factor, ARF1, ADP-ribosylation factor 1, ERK1/2, extracellular signal–regulated protein kinases 1 and 2, FBS, fetal bovine serum, FKBP, FK506-binding protein, FRB, FKBP–rapamycin binding, GA, Golgi apparatus, GCA, golgicide A, GEF, guanine nucleotide exchange factor, GPCR, G protein–coupled receptor, GST, glutathione-*S*-transferase, HEK293, human embryonic kidney 293 cells, MAPK, mitogen-activated protein kinase, PM, plasma membrane, RTK, receptor tyrosine kinase, SDF1α, stromal cell–derived factor 1α, sgRNA, single-guide RNA

## Abstract

After activation of G protein–coupled receptors, G protein βγ dimers may translocate from the plasma membrane to the Golgi apparatus (GA). We recently report that this translocation activates extracellular signal–regulated protein kinases 1 and 2 (ERK1/2) *via* PI3Kγ; however, how Gβγ–PI3Kγ activates the ERK1/2 pathway is unclear. Here, we demonstrate that chemokine receptor CXCR4 activates ADP-ribosylation factor 1 (ARF1), a small GTPase important for vesicle-mediated membrane trafficking. This activation is blocked by CRISPR–Cas9-mediated knockout of the GA-translocating Gγ9 subunit. Inducible targeting of different Gβγ dimers to the GA can directly activate ARF1. CXCR4 activation and constitutive Gβγ recruitment to the GA also enhance ARF1 translocation to the GA. We further demonstrate that pharmacological inhibition and CRISPR–Cas9-mediated knockout of PI3Kγ markedly inhibit CXCR4-mediated and Gβγ translocation–mediated ARF1 activation. We also show that depletion of ARF1 by siRNA and CRISPR–Cas9 and inhibition of GA-localized ARF1 activation abolish ERK1/2 activation by CXCR4 and Gβγ translocation to the GA and suppress prostate cancer PC3 cell migration and invasion. Collectively, our data reveal a novel function for Gβγ translocation to the GA to activate ARF1 and identify GA-localized ARF1 as an effector acting downstream of Gβγ–PI3Kγ to spatiotemporally regulate G protein–coupled receptor signaling to mitogen-activated protein kinases.

G protein–coupled receptors (GPCRs) constitute the largest and most structurally diverse superfamily of membrane signaling proteins and modulate a wide variety of fundamental cellular processes (1, 2). Although the functions of GPCRs are mainly mediated through typical signaling cascades at the plasma membrane (PM) to activate cognate heterotrimeric G proteins, arrestins, and other signaling molecules, recent studies have demonstrated that the activation of PM GPCRs can induce the translocation of Gβγ dimers from the PM to the Golgi apparatus (GA) and the translocation efficiency is mainly determined by Gγ subunits, particularly their C termini (3, 4, 5, 6, 7, 8, 9). Among 12 Gγ subunits, Gγ9 is a unique GA-translocating subunit, both in terms of translocation rate and translocation magnitude. The GA-localized Gβγ complex can activate phospholipase C (10, 11) and protein kinase D (12, 13) and regulate post-Golgi trafficking (13, 14, 15), Golgi structure (12, 15, 16), insulin secretion (16), and cardiomyocyte hypertrophic growth (10). We recently demonstrate that Gβγ translocation to the GA induced by both CXCR4 activation and direct recruitment strongly activates extracellular signal–regulated kinase 1 and 2 (ERK1/2), two members of the mitogen-activated protein kinase (MAPK) family, and this function of Gβγ is mediated through PI3Kγ (9).

ADP-ribosylation factors (ARFs) belong to the superfamily of Ras-related small GTPases. Among six ARFs (ARF1–6) identified in mammalian cells, ARF1 is the best-studied and well-understood member. ARF1 localizes mainly throughout the GA and in the cytoplasm but also at the endoplasmic reticulum–Golgi intermediate compartment, *trans*-Golgi network, endosomes, and the PM. ARF1 is best known for its functions in maintaining the structure and function of the GA and in vesicular trafficking, particularly in the formation of coat protein complex I– and clathrin-coated vesicles, which mediate cargo transport between the endoplasmic reticulum and the GA and between the *trans*-Golgi network and endosomes, respectively (17, 18).

As with all other small GTPases, ARF1 undergoes cycling between the active GTP-bound and inactive GDP-bound conformations, which is under control by guanine nucleotide exchange factors (GEFs) and GTPase-activating proteins. Among 15 ARF–GEFs encoded in the human genome, three high–molecular weight GEFs (GBF1, BIG1, and BIG2), which mainly localize at the GA, and four low–molecular weight GEFs (cytohesin 1–4), which largely express at the PM and endosomes, are able to activate ARF1 (17, 18).

Here, we demonstrate that Gβγ translocation to the GA strongly activates ARF1 *via* PI3Kγ and that GA-localized ARF1 mediates ERK1/2 activation by chemokine receptor CXCR4 activation and Gβγ translocation to the GA. These data provide important insights into how activation of GA-localized ARF1 by Gβγ translocation to the GA spatially and temporally controls GPCR signaling to the MAPK pathway.

## Results

### ARF1 activation by CXCR4 and the role of Gβγ translocation to the GA

**Figure 1:**
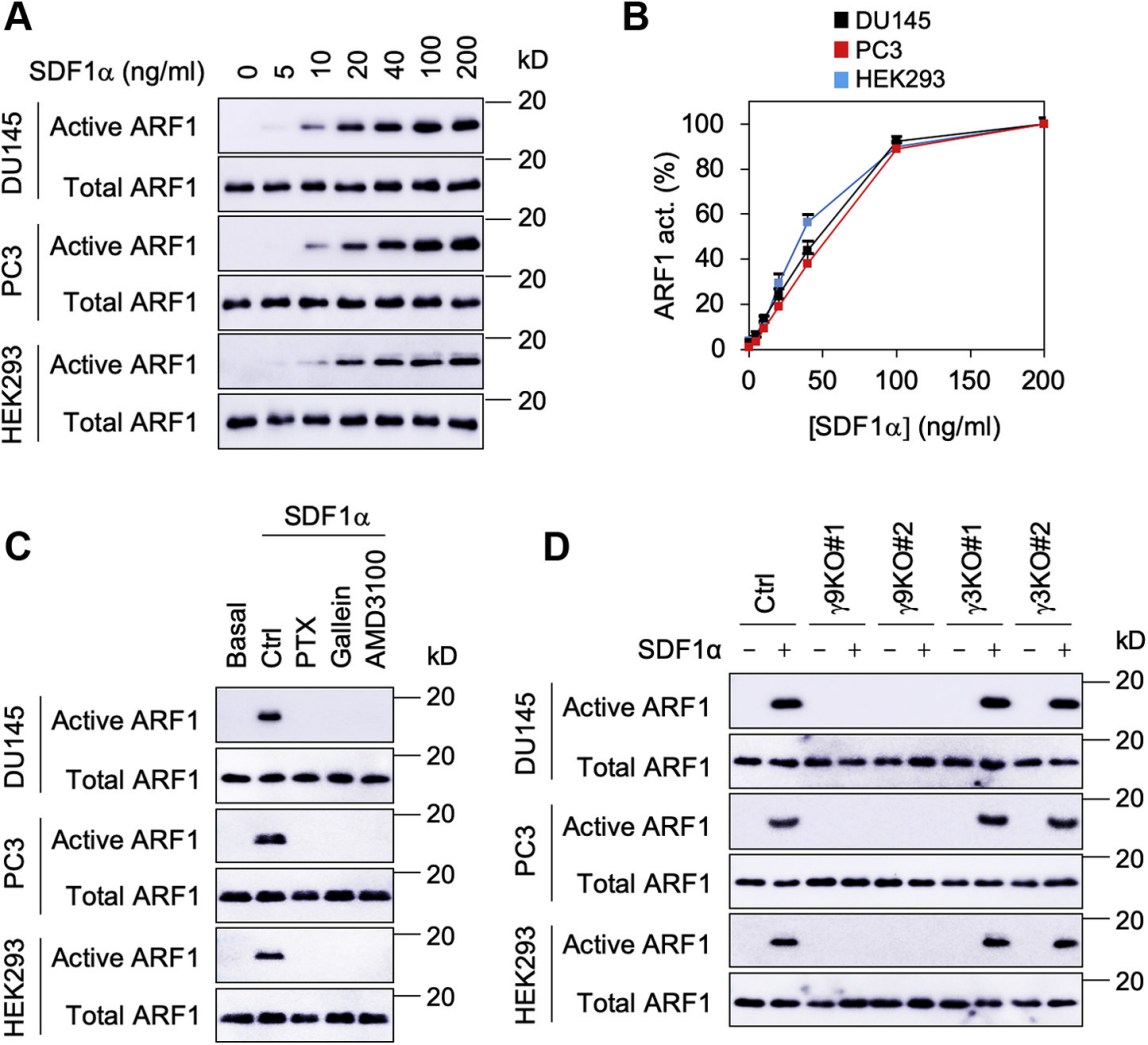
**ARF1 activation by CXCR4 and the role of Gγ9 subunit.***A*, SDF1α dose dependently activated ARF1. The cells cultured on 6-well dishes were starved and then stimulated with different concentrations of SDF1α (0–200 ng/ml). ARF1 activation was measured by GST fusion protein pulldown assays. *B*, quantitative data shown in *A*. ARF1 activation by SDF1α at 200 ng/ml was defined as 100%. *C*, effect of PTX, gallein, and AMD3100 on ARF1 activation by SDF1α. The cells were incubated with PTX (100 ng/ml for 16 h), gallein (10 μM for 30 min), and AMD3100 (100 μM for 1 h) before SDF1α stimulation. *D*, effect of CRISPR–Cas9-mediated knockout of Gγ3 and Gγ9 on ARF1 activation by SDF1α. The quantitative data are presented as means ± SD (n = 3). The Western blots shown in each panel are representatives of at least three experiments. ARF1, ADP-ribosylation factor 1; GST, glutathione-*S*-transferase; PTX, pertussis toxin; SDF1α, stromal cell–derived factor 1α.

We first determined if activation of endogenous CXCR4 by stromal cell–derived factor 1α (SDF1α) could activate ARF1 in human androgen–insensitive prostate cancer (DU145 and PC3) cells and human embryonic kidney 293 (HEK293) cells. As measured in glutathione-*S*-transferase (GST) fusion protein pulldown assays, ARF1 activation by SDF1α was in a dose-dependent fashion in all three cell types and the EC_50_ values were 6.3 ± 0.43, 7.0 ± 0.35, and 4.1 ± 0.52 nM in DU145, PC3, and HEK293 cells, respectively (Fig. 1, *A* and *B*).

We next defined the role of G proteins in ARF1 activation by SDF1α. Treatment with pertussis toxin and the Gβγ-specific inhibitor gallein markedly inhibited ARF1 activation by SDF1α (Fig. 1*C*), suggestive of a role of Gβγ dimers, but not Gα subunits, in ARF1 activation by SDF1α. In addition, treatment with AMD3100, a specific CXCR4 antagonist, abolished ARF1 activation by SDF1α (Fig. 1*C*).

To determine the role of Gβγ translocation onto the GA in ARF1 activation, we determined the effects of CRISPR–Cas9-mediated knockout of Gγ9 and Gγ3 on ARF1 activation by SDF1α. Previous studies have shown that Gγ9 is the most GA-translocating Gγ subunit, whereas Gγ3 is the least GA-translocating Gγ subunit in DU145, PC3, and HEK293 cells (9). Gγ9 knockout completely abolished, whereas Gγ3 knockout did not affect ARF1 activation by SDF1α (Fig. 1*D*).

**Figure 2:**
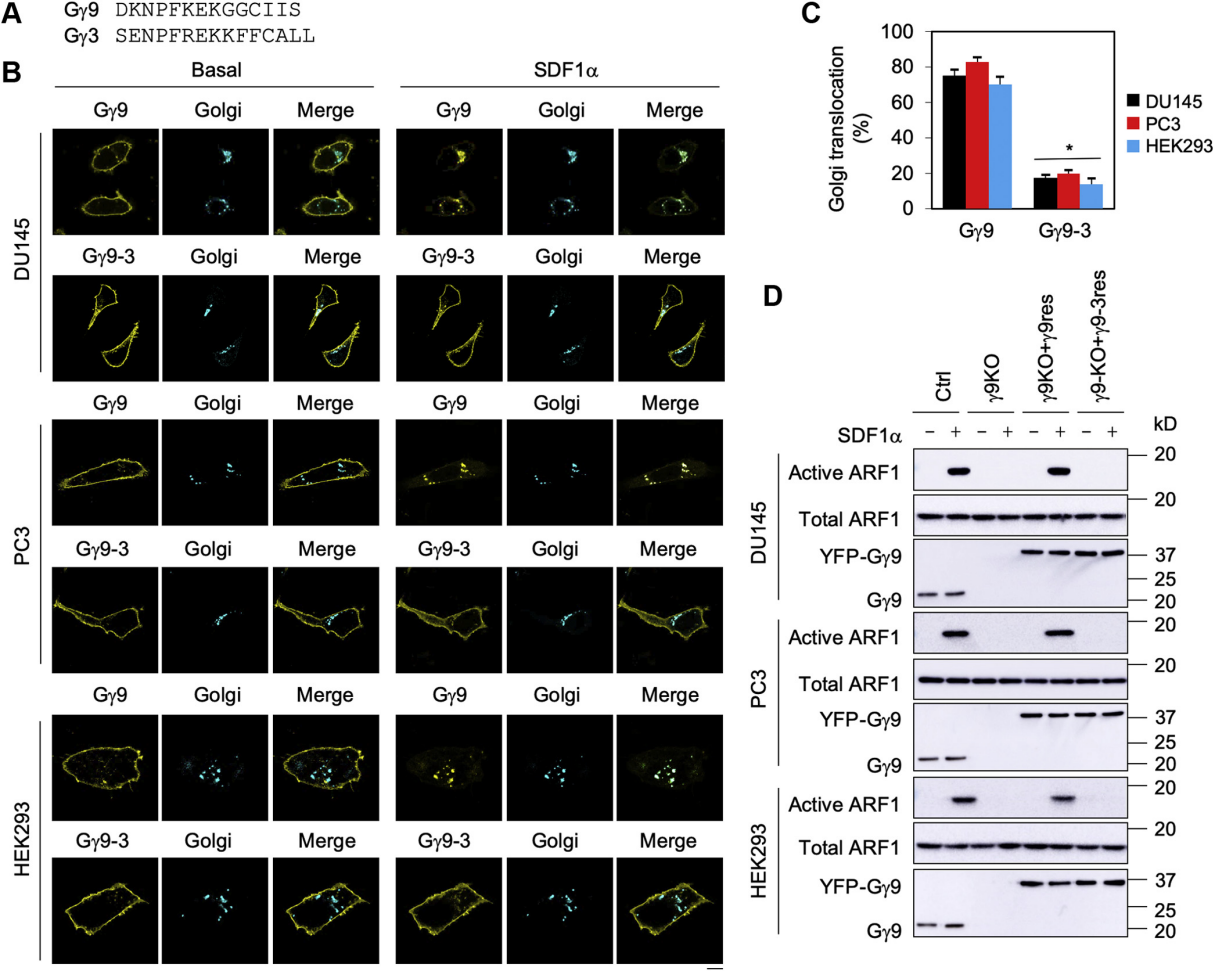
**Translocation of Gγ9 and Gγ9-3 from the PM to the GA and their abilities to rescue ARF1 activation in Gγ9 knockout cells.***A*, the C-terminal sequences of Gγ9 and Gγ3. *B*, translocation of Gγ9 and Gγ9-3 from the PM to the GA. The cells were cultured on coverslips and transfected with YFP-Gγ9 or YFP-Gγ9-3 together with Gβ1, Gαi1, and pmTurquoise2-Golgi (500 ng each). After starvation, the cells were stimulated with SDF1α at 1 μg/ml, and the images shown are obtained after stimulation for 10 and 55 s in cells expressing Gγ9 and Gγ9-3, respectively. *C*, quantification of GA translocation of Gγ9 and Gγ9-3 in complex with Gβ1 in response to SDF1α stimulation. The increase in the YFP signal at the GA after SDF1α stimulation was considered as Gβγ translocation to the GA. The data were expressed as relative to total YFP signal in cell. *D*, rescue of ARF1 activation in response to SDF1α stimulation by transient expression of sgRNA-resistant Gγ9, but not Gγ9-3, in Gγ9 knockout cells. Gγ9 knockout cells cultured on 6-well dishes were transfected with sgRNA-resistant Gγ9 (Gγ9res) or Gγ9-3 (Gγ9-3res) plasmids. After starvation, the cells were stimulated with SDF1α at 1 μg/ml for 5 min. ARF1 activation was measured by GST fusion protein pulldown assays. In each cell line, *bottom panel* shows the expression of endogenous Gγ9 and exogenous YFP-Gγ9 detected by using Gγ9 antibodies. The images shown in *B* and *D* are representatives of at least three experiments. The quantitative data in *C* are presented as means ± SD (n = 5–8). ∗*p* < 0.05 *versus* Gγ9. The scale bar represents 10 μm. ARF1, ADP-ribosylation factor 1; GA, Golgi apparatus; GST, glutathione-*S*-transferase; PM, plasma membrane; SDF1α, stromal cell–derived factor 1α; sgRNA, single-guide RNA.

To further confirm the role of Gβγ translocation to the GA in ARF1 activation, we compared the abilities to rescue ARF1 activation in Gγ9 knockout cells by single-guide RNA (sgRNA)–resistant Gγ9 and its mutant Gγ9-3 in which the C-terminal 14 residues of Gγ9 were substituted with the C-terminal 15 residues of Gγ3 (Fig. 2*A*). Consistent with previous studies (6, 9), Gγ9 robustly translocated from the PM to the GA with greater than 70% efficacy, whereas Gγ9-3 remained largely at the PM with only 15% being translocated to the GA after SDF1α stimulation in DU145, PC3, and HEK293 cells (Fig. 2, *B* and *C*). As expected, transient expression of sgRNA-resistant Gγ9 successfully rescued ARF1 activation by SDF1α in Gγ9 knockout cells. In contrast, expression of GA translocation defective mutant Gγ9-3 was ineffective (Fig. 2*D*). These data strongly suggest that ARF1 activation by SDF1α is mediated through Gβγ translocation to the GA.

### Inducible Gβγ translocation to the GA constitutively activates ARF1

**Figure 3:**
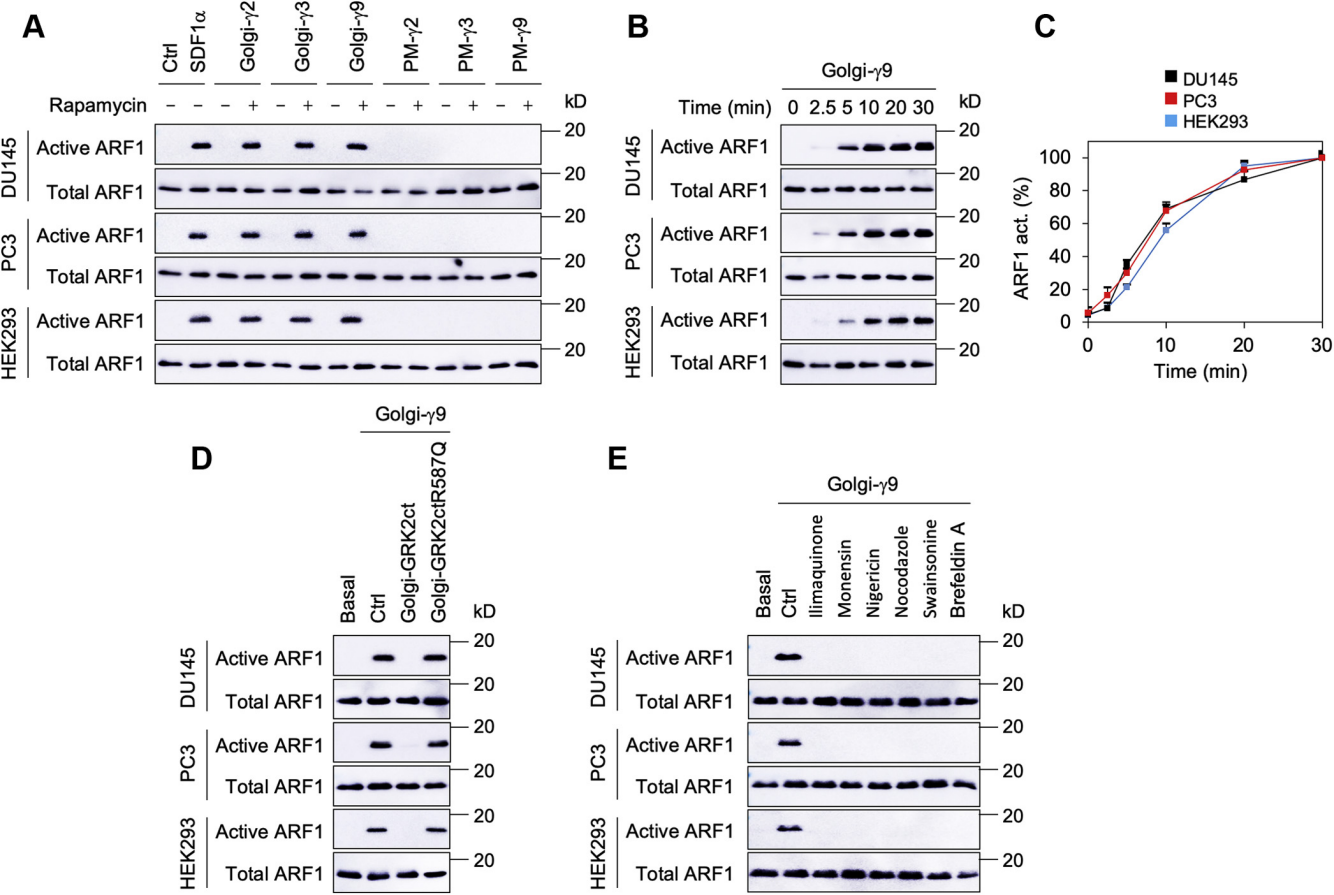
**Targeting of Gβγ dimers to the GA, but not the PM, constitutively activates ARF1.***A*, inducible expression of Gγ2, Gγ3, and Gγ9 at the GA, but not at the PM, activated ARF1. The cells were transfected with individual FRB-Gγ and Gβ1, together with Golgi-FKBP or PM–FKBP (500 ng each), and then induced with rapamycin at 1 μM for 30 min. SDF1α simulation was used as a positive control. *B*, time courses of ARF1 activation by Golgi-Gγ9. *C*, quantitative data shown in *B*. ARF1 activation at 30 min was defined as 100%. *D*, effect of GRK2ct on ARF1 activation by Golgi-Gγ9. The cells were transfected with FRB-Gγ9, Golgi-FKBP, Gβ1, and Golgi-GRK2ct or Golgi-GRK2ctR587Q (500 ng each) and then incubated with rapamycin for 30 min. *E*, effect of Golgi disruptors on ARF1 activation by Golgi-Gγ9. The cells transfected with FRB-Gγ9, Gβ1, and Golgi-FKBP were treated with ilimaquinone (10 μM), monensin (5 μM), nigericin (2 μM), nocodazole (10 μM), swainsonine (5 μM), and brefeldin A (3 μM) for 30 min before incubation with rapamycin. The data in *C* are presented as means ± SD (n = 3). The Western blots shown are representatives of at least three experiments. ARF1, ADP-ribosylation factor 1; FKBP, FK506-binding protein; FRB, FKBP–rapamycin binding; GA, Golgi apparatus; PM, plasma membrane; SDF1α, stromal cell–derived factor 1α.

We next determined the effect of direct recruitment of Gβγ dimers onto the GA and PM on ARF1 activation by using the well-characterized rapamycin-inducible translocation system in which GA- and PM-targeting peptides were fused to FK506-binding protein (FKBP), whereas cytosolic Gγ subunits were fused to the FKBP–rapamycin binding (FRB) domain (9, 10, 13). Rapamycin-induced GA recruitment of Gγ2, Gγ3, or Gγ9 subunits (Golgi-Gγ), each in complex with Gβ1, activated ARF1 in DU145, PC3, and HEK293 cells. In contrast, their recruitment onto the PM (PM-Gγ) had no clear effect on ARF1 activation (Fig. 3*A*). Rapamycin incubation to induce Gγ9 translocation to the GA activated ARF1 in a time-dependent manner (Fig. 3, *B* and *C*). ARF1 activation by Golgi-Gγ9 was strongly blocked by coexpression of GA-localized GRK2ct, which binds Gβγ dimers, but not GRK2ctR587Q mutant, which lacks the Gβγ-binding ability (Fig. 3*D*). ARF1 activation by Golgi-Gγ9 was also inhibited by treatment with all Golgi disruptors tested, including ilimaquinone, monensin, nigericin, nocodazole, swainsonine, and brefeldin A (Fig. 3*E*). These data demonstrate that constitutive targeting of different Gβγ dimers to the GA can directly activate ARF1.

### CXCR4 activation and constitutive Gβγ translocation to the GA enhance ARF1 recruitment to the GA

**Figure 4:**
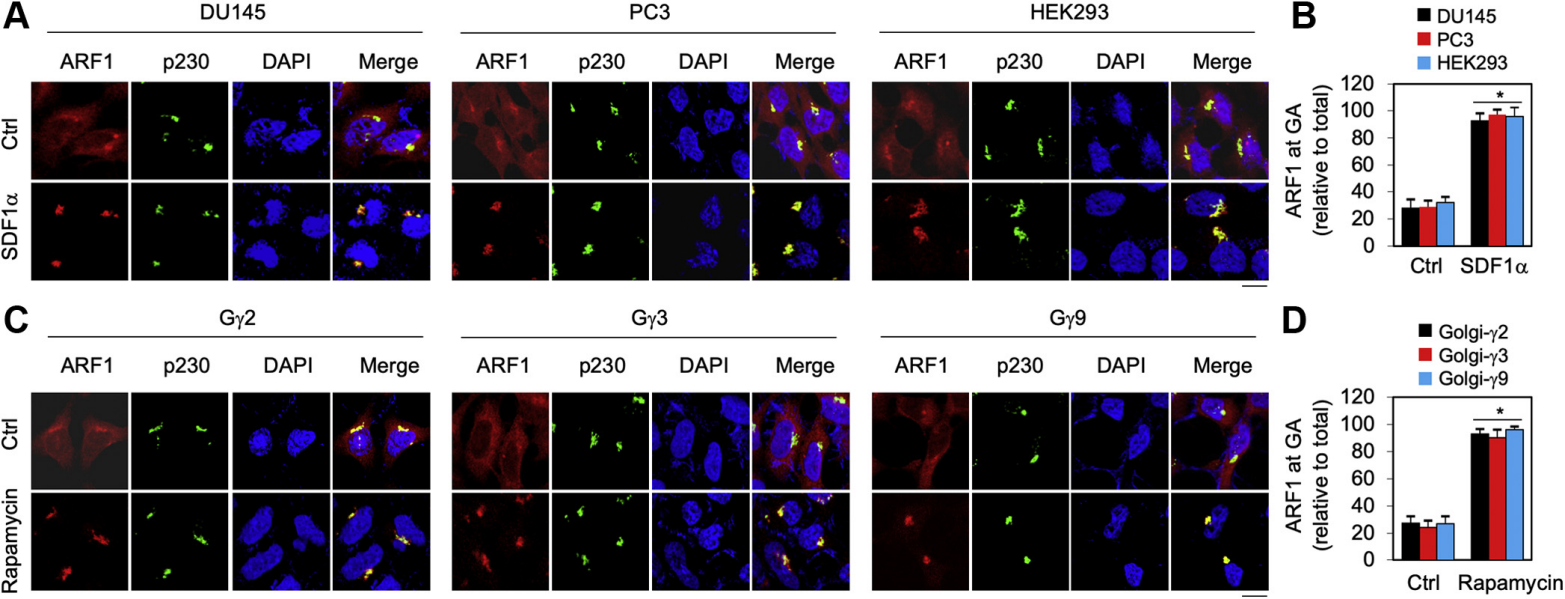
**ARF1 recruitment to the GA in response to SDF1α stimulation and inducible Gβγ translocation to the GA.***A*, ARF1 translocation to the GA after SDF1α stimulation in DU145, PC3, and HEK293 cells. The cells cultured on coverslip were stimulated with SDF1α at 200 ng/ml for 5 min and then stained with antibodies against ARF1 and p230. *B*, quantification of ARF1 expression at the GA by using p230 as a GA marker. *C*, ARF1 translocation to the GA induced by Gβγ translocation to the GA in DU145 cells. The cells were transfected with individual FRB-Gγ, Gβ1, and Golgi-FKBP and incubated with rapamycin at 1 μM for 30 min. *D*, quantitative data shown in *C*. The data in *B* and *D* are presented as means ± SD (n = 55–65 cells in three experiments). ∗*p* < 0.05 *versus* respective control. The scale bars represent 10 μm. ARF1, ADP-ribosylation factor 1; FRB, FKBP–rapamycin binding; GA, Golgi apparatus; HEK293, human embryonic kidney 293 cells; SDF1α, stromal cell–derived factor 1α.

It has been well demonstrated that, similar to many other small GTPases, the active form of ARF1 is membrane bound, whereas the inactive form of ARF1 is cytosolic (19, 20, 21). As such, we used confocal microscopy to define the effect of CXCR4 activation and constitutive Gβγ targeting to the GA on the recruitment of endogenous ARF1 to the GA, which presumably reflects its activation. At the basal level, ARF1 was mainly expressed in the cytoplasm with less than one-third of total ARF1 at the GA in DU145, PC3, and HEK293 cells as quantified by using p230 as a GA marker. After SDF1α stimulation for 5 min, more than 90% of total ARF1 translocated to the GA (Fig. 4, *A* and *B*). Similar to SDF1α stimulation, rapamycin-induced GA recruitment of Gγ2, Gγ3, or Gγ9 subunits in complex with Gβ1 dramatically increased ARF1 localization at the GA in PC3 cells (Fig. 4, *C* and *D*). In contrast, rapamycin-induced PM recruitment of Gγ9 in complex with Gβ1 did not affect the subcellular distribution of ARF1 in DU145, PC3, and HEK293 cells (Fig. S1). These data further demonstrate that CXCR4 activation and Gβγ translocation to the GA are able to activate ARF1.

### PI3Kγ mediates ARF1 activation by CXCR4 and Gβγ translocation to the GA

**Figure 5:**
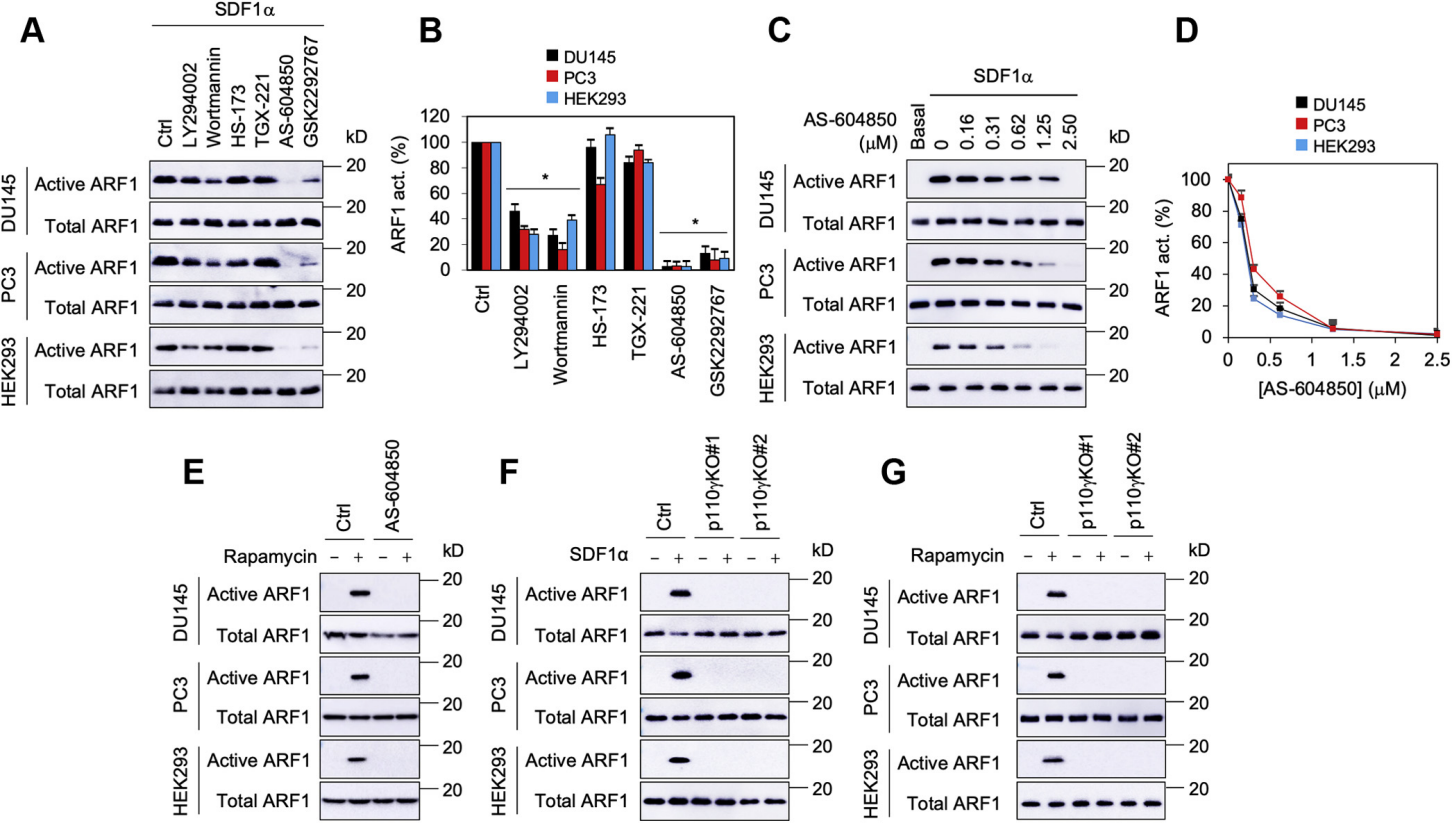
**Effects of pharmacological inhibition and knockout of PI3Kγ on ARF1 activation by SDF1α and Golgi-Gγ9.***A*, effect of PI3K inhibitors on ARF1 activation by SDF1α. The cells were incubated with LY294002 (50 μM), wortmannin (10 μM), HS-173 (0.1 μM), TGX-221 (0.5 μM), AS-604850 (2.5 μM), or GSK2292767 (0.5 μM) for 6 h before stimulation with SDF1α at 200 ng/ml for 5 min. *B*, quantitative data shown in *A*. *C*, dose-dependent effect of AS-604850 on ARF1 activation by SDF1α. *D*, quantitative data shown in *C*. ARF1 activation by SDF1α without AS-604850 was defined as 100%. *E*, effect of AS-604850 on ARF1 activation by Golgi-Gγ9. The cells were transfected with FRB-Gγ9, Gβ1, and Golgi-FKBP and treated with AS-604850 at 2.5 μM for 6 h before incubation with rapamycin at 1 μM for 30 min. *F*, effect of CRISPR–Cas9-mediated knockout of p110γ on ARF1 activation by SDF1α stimulation at 200 ng/ml for 5 min. *G*, effect of p110γ knockout on ARF1 activation by Golgi-Gγ9. The quantitative data are presented as means ± SD (n = 3). The Western blots shown in each panel are representatives of at least three experiments. ∗*p* < 0.05 *versus* respective control. ARF1, ADP-ribosylation factor 1; FKBP, FK506-binding protein; FRB, FKBP–rapamycin binding; SDF1α, stromal cell–derived factor 1α.

As PI3Kγ is a well-known downstream effector of Gβγ and mediates ERK1/2 activation by Gβγ translocation to the GA (9), we determined its role in ARF1 activation by CXCR4 and Golgi-Gγ9. Inhibition of PI3K activation by LY294002, wortmannin (two common PI3K inhibitors), AS-604850 (a PI3Kγ inhibitor) and GSK2292767 (a PI3Kδ inhibitor), but not with HS-173 (a PI3Kα inhibitor) and TGX-221 (a PI3Kβ inhibitor), significantly inhibited ARF1 activation by SDF1α (Fig. 5, *A* and *B*). The inhibitory action of AS-604850 was in a dose-dependent fashion, and the IC_50_ values were 0.23 ± 0.08, 0.31 ± 0.06, and 0.21 ± 0.04 μM in DU145, PC3, and HEK293 cells, respectively (Fig. 5, *C* and *D*). Inhibition of PI3Kγ activation by AS-604850 also reduced ARF1 activation induced by Golgi-Gγ9 (Fig. 5*E*). CRISPR–Cas9-mediated knockout of p110γ, the catalytic subunit of PI3Kγ, completely abolished ARF1 activation by SDF1α stimulation (Fig. 5*F*) and Golgi-Gγ9 (Fig. 5*G*). These data demonstrate that PI3Kγ is an important element in ARF1 activation by CXCR4 and Gβγ translocation to the GA.

### Depletion of ARF1 abolishes ERK1/2 activation by CXCR4 and Gβγ translocation to the GA

**Figure 6:**
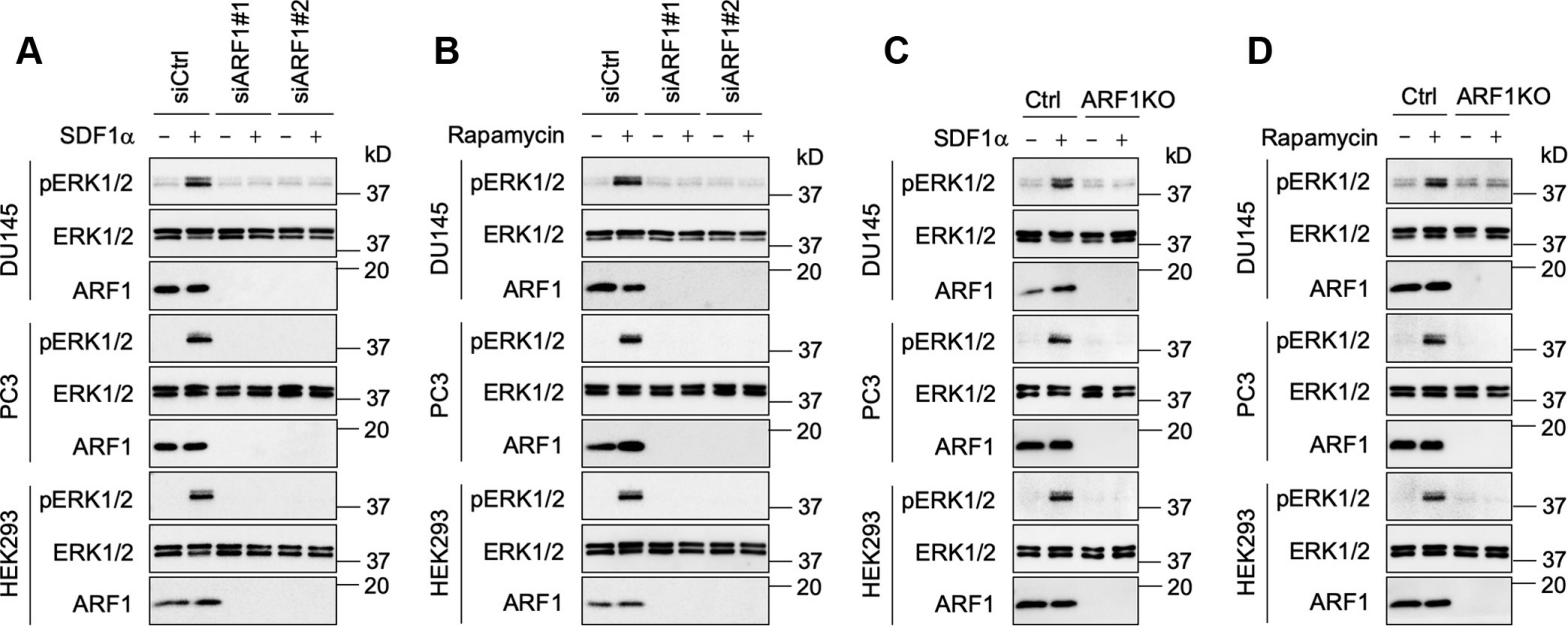
**Depletion of ARF1 by siRNA and CRISPR–Cas9 abolishes ERK1/2 activation by SDF1α and Golgi-Gγ9.***A*, effect of siRNA-mediated ARF1 knockdown on ERK1/2 activation by SDF1α stimulation at 200 ng/ml for 5 min. *B*, effect of ARF1 knockdown by siRNA on ERK1/2 activation by Golgi-Gγ9. The cells were transfected with FRB-γ9, Gβ1, and Golgi-FKBP (500 ng each) and then incubated with rapamycin for 30 min. *C*, effect of CRISPR–Cas9-mediated depletion of ARF1 on ERK1/2 activation by SDF1α. *D*, effect of ARF1 depletion by CRISPR–Cas9 on ERK1/2 activation by Golgi-Gγ9. The Western blots shown in each panel are representatives of at least three experiments. ARF1, ADP-ribosylation factor 1; ERK1/2, extracellular signal–regulated protein kinases 1 and 2; FKBP, FK506-binding protein; FRB, FKBP–rapamycin binding; SDF1α, stromal cell–derived factor 1α.

Our preceding data have shown that, similar to ERK1/2 activation, Gβγ translocation to the GA activates ARF1 *via* PI3Kγ, suggesting that ARF1 may function as a downstream effector of Gβγ–PI3Kγ to activate ERK1/2. To test this possibility, we first determined the effect of ARF1 depletion by siRNA and CRISPR–Cas9 on ERK1/2 activation by CXCR4 and Gβγ translocation to the GA. ARF1 knockdown by siRNA substantially inhibited ERK1/2 activation by SDF1α (Fig. 6*A*) and Golgi-Gγ9 (Fig. 6*B*) in DU145, PC3, and HEK293 cells. ARF1 knockout by CRISPR–Cas9 also remarkably inhibited ERK1/2 activation by SDF1α stimulation (Fig. 6*C*) and Golgi-Gγ9 (Fig. 6*D*). These data demonstrate that the normal expression of endogenous ARF1 is directly linked to ERK1/2 activation by CXCR4 and Gβγ translocation to the GA.

To exclude the possibility that the inhibitory effect of ARF1 depletion on ERK1/2 activation by SDF1α stimulation is partially attributable to its effect on the cell surface expression of CXCR4, we visualized the subcellular localization of CXCR4 in ARF1-depleted cells by confocal microscopy. ARF1 depletion by siRNA and CRISPR–Cas9 did not affect the surface presentation of CXCR4 in all three cell types tested (Fig. S2, A and B).

### Inhibition of GA-localized ARF1 attenuates ERK1/2 activation by CXCR4 and Gβγ translocation to the GA

**Figure 7:**
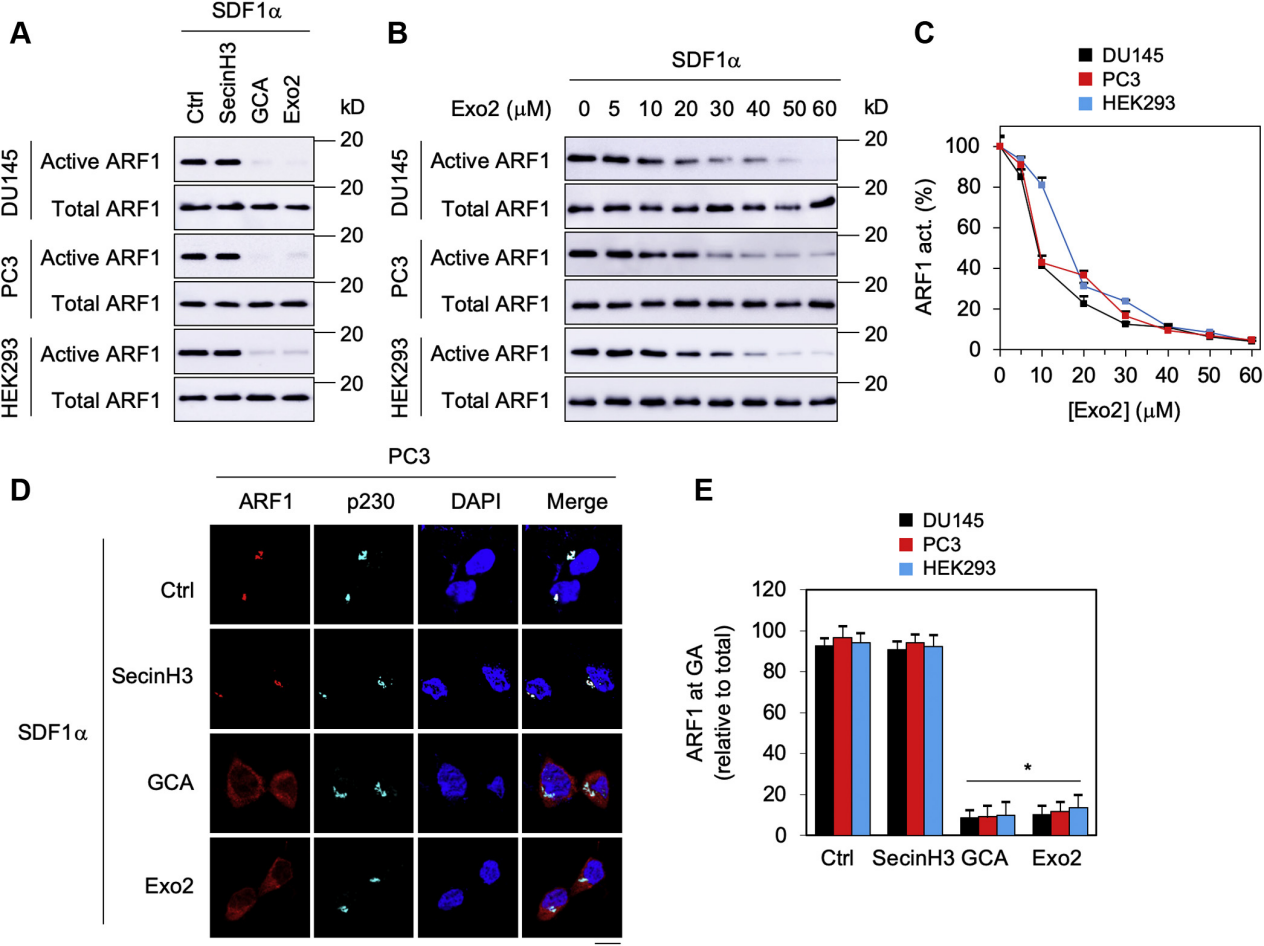
**Effects of ARF1 inhibitors on the activation and GA recruitment of ARF1 induced by SDF1α.***A*, effects of ARF1 inhibitors on ARF1 activation. The cells were treated with secinH3 (100 μM), GCA (30 μM), or Exo2 (60 μM) for 30 min before stimulation with SDF1α at 200 ng/ml for 5 min. *B*, dose-dependent effect of Exo2 on ARF1 activation by SDF1α. *C*, quantitative data shown in *B* (n = 3). *D*, effects of ARF1 inhibitors on ARF1 localization at the GA. The cells were treated with individual inhibitors before SDF1α stimulation as mentioned previously, and ARF1 localization at the GA was visualized by confocal imaging following staining with antibodies against ARF1 and p230. *E*, quantitative data showing ARF1 expression at the GA relative to its total expression (n = 50 cells in three experiments). The quantitative data shown in *C* and *E* are presented as means ± SD. The images shown in each panel are representatives of at least three experiments. ∗*p* < 0.05 *versus* respective control. The scale bar represents 10 μm. ARF1, ADP-ribosylation factor 1; GA, Golgi apparatus; GCA, golgicide A; SDF1α, stromal cell–derived factor 1α.

We next determined the effects of inhibiting ARF1 on ERK1/2 activation by CXCR4 and Gβγ translocation to the GA. For this purpose, three small molecule inhibitors, including golgicide A (GCA), Exo2, and secinH3, were used. Whereas GCA and Exo2 selectively inhibit Golgi-associated ARF–GEFs, secinH3 inhibits only PM-associated ARF–GEFs (22, 23, 24, 25). Treatment with these GEF inhibitors for 30 min did not affect the surface expression of CXCR4 (Fig. S2C). Treatment with GCA and Exo2, but not secinH3, dramatically attenuated ARF1 activation by SDF1α in DU145, PC3, and HEK293 cells (Fig. 7*A*), suggesting that activated ARF1 is mainly localized on the GA in these cells. Inhibition of ARF1 activation by Exo2 was in a dose-dependent fashion, and the IC_50_ values were 8.7 ± 0.6, 9.2 ± 0.3, and 16.1 ± 0.8 μM in DU145, PC3, and HEK293 cells, respectively (Fig. 7, *B* and *C*). Similarly, treatment with GCA and Exo2, but not secinH3, strongly attenuated ARF1 localization at the GA in response to SDF1α stimulation in all three cell lines studied (Fig. 7, *D* and *E* and Fig. S3).

**Figure 8:**
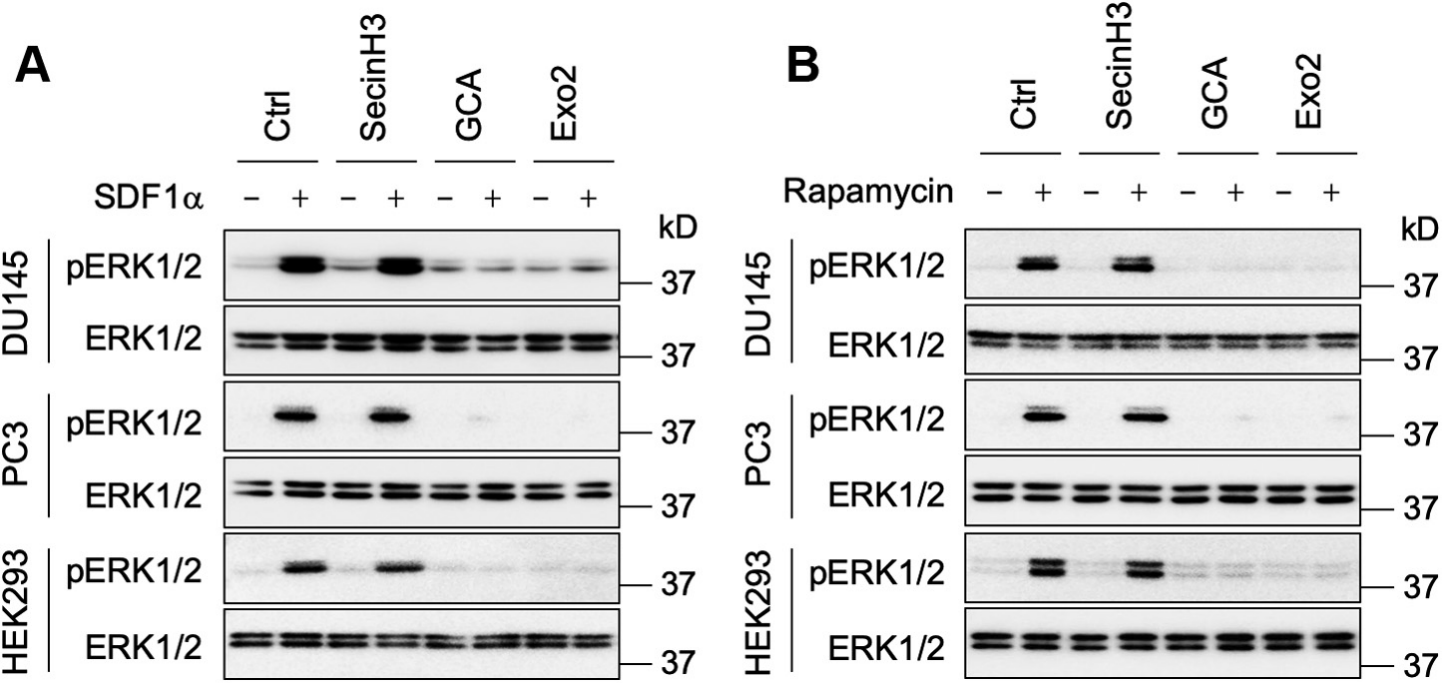
**Effects of ARF1 inhibitors on ERK1/2 activation by SDF1α and Golgi-Gγ9.***A*, effect of ARF1 inhibitors on ERK1/2 activation by SDF1α. The cells were treated with secinH3 (100 μM), GCA (30 μM), or Exo2 (60 μM) for 30 min before stimulation with SDF1α at 200 ng/ml for 5 min. *B*, effect of ARF1 inhibitors on ERK1/2 activation by Golgi-Gγ9. The cells were transfected with FRB-γ9, Gβ1, and Golgi-FKBP (500 ng each) and then incubated with individual inhibitors before incubation with rapamycin for 30 min. The Western blots shown in each panel are representatives of at least three experiments. ARF1, ADP-ribosylation factor 1; ERK1/2, extracellular signal–regulated protein kinases 1 and 2; FKBP, FK506-binding protein; FRB, FKBP–rapamycin binding; GCA, golgicide A; SDF1α, stromal cell–derived factor 1α.

In parallel with their abilities to inhibit ARF1 activation, GCA and Exo2 almost completely abolished ERK1/2 activation by SDF1α (Fig. 8*A*). Similarly, treatment with GCA and Exo2 markedly inhibited ERK1/2 activation by inducible Gβγ translocation to the GA (Fig. 8*B*). In contrast, treatment with secinH3 had no effect on ERK1/2 by SDF1α and forced Gβγ recruitment to the GA (Fig. 8, *A* and *B*). These data suggest that GA-localized ARF1 activation is an important event for ERK1/2 activation by CXCR4 and Gβγ translocation to the GA.

### Inhibition and depletion of ARF1 suppress prostate cancer cell migration and invasion

**Figure 9:**
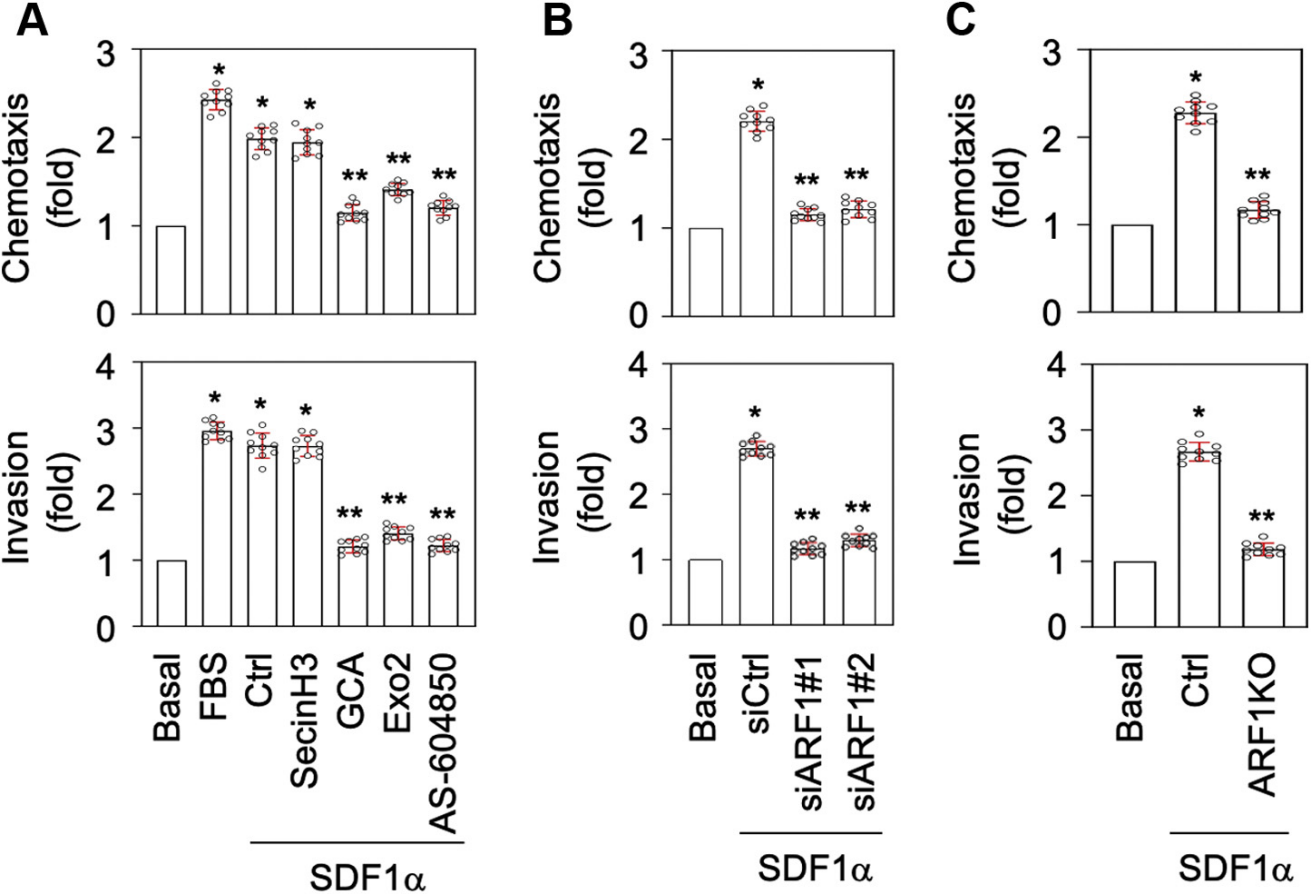
**Inhibition and depletion of ARF1 suppress PC3 migration and invasion induced by SDF1α.***A*, inhibition of PC3 migration and invasion by ARF1 and PI3Kγ inhibitors as measured in transwell assays. PC3 cells were treated with SDF1α at 1 μg/ml together with secinH3 (100 μM), GCA (30 μM), Exo2 (60 μM), or AA-604850 (2.5 μM) for 48 h. Stimulation with FBS at 10% was used as a positive control. *B*, inhibition of PC3 migration and invasion by siRNA-mediated ARF1 knockdown. PC3 cells were transfected with control or ARF1 siRNA and then treated with SDF1α for 48 h. *C*, inhibition of PC3 migration and invasion by CRISPR–Cas9-mediated depletion of ARF1. PC3 cells were transfected with control or ARF1 knockout plasmids and then treated with SDF1α for 48 h. The quantitative data are presented as means ± SD (n = 10). ∗ and ∗∗*p* < 0.005 *versus* basal and control, respectively. ARF1, ADP-ribosylation factor 1; FBS, fetal bovine serum; GCA, golgicide A; SDF1α, stromal cell–derived factor 1α.

Although ARF1 is described to regulate a number of cellular processes in response to activation with receptor tyrosine kinases (RTKs) (17, 18), its roles in GPCR-mediated cell functions remain poorly defined. As such, we determined the effect of ARF1 inhibition and depletion on cell migration and invasion in response to SDF1α stimulation using PC3 cells as models in *in vitro* transwell assays. Stimulation with fetal bovine serum (FBS) was used as a positive control. As expected, stimulation with SDF1α strongly enhanced PC3 cell migration and invasion, which were significantly inhibited by treatment with GCA, Exo2, and AS-604850, but not secinH3 (Fig. 9*A*). ARF1 depletion by siRNA (Fig. 9*B*) and CRISPR–Cas9 (Fig. 9*C*) also greatly suppressed PC3 cell migration and invasion in response to SDF1α stimulation. These data demonstrate that the activation of ARF1, specifically at the GA, directly controls prostate cancer cell migration and invasion in response to CXCR4 activation.

## Discussion

In this study, we first identify a novel function of Gβγ translocation from the PM to the GA to activate the small GTPase ARF1 in three different cells. This function of Gβγ translocation to the GA is strongly supported by the following series of experiments, which use GST fusion protein pulldown assays to biochemically quantify the active form of ARF1. First, knockout of the most GA-translocating Gγ9 subunit, but not the least GA-translocating Gγ3 subunit, abolishes ARF1 activation by CXCR4. Second, wildtype Gγ9, but not its mutant Gγ9-3 defective in GA translocation, is able to rescue ARF1 activation by CXCR4 in Gγ9 knockout cells. Third, chemically induced GA translocation of different Gβγ dimers containing Gγ2, Gγ3, or Gγ9, all constitutively activates ARF1, suggesting that different Gβγ combinations, once expressed at the GA, can activate ARF1. Fourth, inhibition of GA-localized Gβγ *via* GRK2ct, as well as GA disruptors, inhibits ARF1 activation by Gβγ translocation to the GA. The function of Gβγ translocation to the GA in activating ARF1 is also supported by confocal imaging showing that constitutive GA translocation of Gβγ dimers, as well as CXCR4 activation, markedly enhances the GA localization of ARF1, which presumably represents its active form. In addition, we have demonstrated that pharmacologic inhibition of PI3Kγ and knockout of its catalytic subunit p110γ block ARF1 activation by CXCR4 and Gβγ translocation to the GA, indicative of a crucial role of PI3Kγ in ARF1 activation, which is consistent with its function in ARF1 activation by formyl peptide receptors in neutrophils (26). Thus, Gβγ translocation to the GA induced by both GPCR activation and direct recruitment is able to activate ARF1, which is mediated through activation of PI3Kγ.

Another important finding of the current study is that GA-localized ARF1 is an essential mediator for ERK1/2 activation by CXCR4 and Gβγ translocation to the GA. This became evident by our data demonstrating that ARF1 depletion by siRNA and CRISPR–Cas9 and pharmacological inhibition of GA-localized ARF1 abolish ERK1/2 activation by CXCR4 and constitutive Gβγ translocation to the GA. This is also strongly supported by our previous studies showing that expression of constitutively active GTP-bound ARF1 mutant, which almost exclusively localizes at the GA, directly activates the Raf–MEK–ERK1/2 pathway in the absence of any extracellular stimuli (19, 27, 28). Altogether, these studies provide strong evidence indicating that ARF1 activation by Gβγ translocation to the GA spatially and temporally regulate GPCR signaling to MAPK, and that the GA provides a platform to compartmentalize the important events involved in GPCR signaling, including the translocation of Gβγ and sequential activation of PI3Kγ, ARF1, and the MAPK pathway.

Our data presented in this article, together with our previous studies, have uncovered a signaling pathway in which GPCR activation at the PM induces Gβγ translocation to the GA where it activates PI3Kγ, which in turn activates ARF1, leading to activation of the MAPK pathway. It is interesting to note that previous studies have shown that ARF1 regulates phospholipase D activation by GPCRs likely through its physical interaction with the receptors, and these interactions presumably occur at the PM (29, 30, 31, 32). Therefore, GPCRs at the PM may use distinct mechanisms to activate ARF1 in different subcellular compartments, which may cause the activation of different signaling pathways (*e.g.*, ARF1 at the GA activates MAPK, whereas ARF1 at the PM activates phospholipase D). Several studies also demonstrate that ARF1 regulates the activation of PI3Kα, type I phosphatidylinositol 4-phosphate 5-kinase, and Rac by RTKs (33, 34, 35, 36, 37, 38, 39, 40, 41, 42), most likely *via* classic PM signaling pathways. As such, to the best of our knowledge, the MAPK activation represents the first defined signal transduction pathway, which is under control by GA-localized ARF1. However, the detailed molecular mechanisms of how PI3Kγ activates ARF1 and how ARF1 activates the MAPK pathway need further investigation.

The MAPK pathway controls a number of fundamental cellular processes under the physiologic and pathologic conditions and is subject to regulation by GPCR activation. As such, the ability of ARF1 to mediate MAPK activation by GPCRs may have broad implications. By using genetic, biochemical, and pharmacologic approaches, ARF1 has been well demonstrated to play roles in cell growth, migration, and invasion in response to RTK activation, which are mediated *via* regulating protein trafficking or activating signaling molecules (28, 37, 42, 43, 44, 45). However, its roles in GPCR-mediated cellular functions are poorly defined. Our data demonstrate that GA-localized ARF1 inhibitors and ARF1 depletion by siRNA and CRISPR–Cas9 remarkably suppress prostate cancer cell migration and invasion induced by activation of CXCR4, an important GPCR involved in prostate cancer metastasis (46, 47). These data suggest that activation of ARF1, particularly its GA-localized pool, by GPCRs *via* Gβγ translocation and PI3Kγ, which leads to the activation of oncogenic MAPK signaling cascades, may have pathophysiological implications in cancer biology. It is worth mentioning that ARF1 is a multifunction GTPase; therefore, in addition to regulating MAPK, its activation by GPCRs and Gβγ translocation to the GA may have other functional consequences. Since ARF1 is best known for its crucial roles in the formation of transport vesicles, GPCR-induced Gβγ translocation to the GA may play a role in membrane trafficking *via* activating ARF1. Indeed, Gβγ translocation was recently demonstrated to regulate protein transport at the level of the GA (13). However, whether this function of GA-localized Gβγ is mediated through ARF1 is unknown.

It is known that almost all GPCRs are able to activate the Raf–MEK–ERK1/2 pathway, and extensive efforts have been made to elucidate the underlying activation mechanisms. Over the past decades, a number of distinct biochemical pathways, as well as many different signaling molecules, have been identified to contribute to GPCR-mediated MAPK activation. Gβγ dimers are well defined to regulate the MAPK activation by GPCRs, particularly those coupled to Gi proteins. This function of Gβγ is generally considered to be restricted at the PM and can be mediated through the activation of RTKs, Src family kinases, and PLCβ, or the recruitment of arrestins to the phosphorylated receptors, leading to the formation of the MAPK activation scaffolds (48, 49, 50, 51). Although our studies have strongly demonstrated that ERK1/2 activation by CXCR4 is almost exclusively mediated through a mechanism involving Gβγ translocation to the GA and activation of PI3Kγ and ARF1, we cannot exclude the possibility that ERK1/2 activation by different GPCRs can occur through distinct mechanisms as described previously.

In summary, the data presented in this article reveal a novel function for Gβγ translocation to the GA to activate ARF1 and identify GA-localized ARF1 as a crucial mediator of CXCR4 signaling to the MAPK pathway. Overall, these data provide important insights into spatiotemporal regulation of the functionality of the GPCR members.

## Experimental procedures

### Materials

Human SDF1α was purchased from PeproTech; UK14304, rapamycin, brefeldin A, monensin, nigericin, swainsonine, and Exo2 were from Sigma–Aldrich; pertussis toxin was from List Biological Laboratories; gallein was from Tocris Bioscience; wortmannin, AS-604850, and GSK2292767 were from ApexBio; TGX-221 and HS-173 were from Adooq Bioscience; secinH3, GCA, nocodazole, ilimaquinone, AMD3100, control siRNA, and siRNA targeting human ARF1, CRISPR–Cas9 control plasmids, and knockout plasmids targeting human ARF1, and antibodies against phospho-ERK1/2 and β-actin were from Santa Cruz Biotechnology; p230 antibodies were from BD Transduction Laboratories; ARF1 antibodies were purchased from Abcam; ERK1/2 antibodies were from Cell Signaling Technology; 12-well inserts and Matrigel matrix were from Corning. All other materials were obtained as described elsewhere (9, 19, 27).

### Plasmid DNA constructs

Golgi-FKBP, PM-FKBP, FRB-Gγ2, and Golgi-GRK2ct plasmids were kindly provided by Drs Alan V. Smrcka and Philip B. Wedegaertner as described (10, 13), YFP-tagged CXCR4 by Adriano Marchese, and Gβ1, Gαi1, and pmTurquoise2-Golgi constructs by Nevin A. Lambert. The YFP-tagged Gγ9 (#36107) and Gγ9-3 (#36074) were obtained from Addgene as described (6). FRB-Gγ3 and FRB-Gγ9 constructs were generated by mutating Cys in the CAAX motif of Gγ3 and Gγ9 into Ser, which were then fused with FRB as described previously (9). Golgi-GRK2ctR587Q mutant was generated by using the QuikChange site-directed mutagenesis kit (Agilent). sgRNA-resistant YFP-Gγ9 rescue plasmid was generated as described previously (9), and the same primers were used to generate sgRNA-resistant YFP-Gγ9-3 plasmid.

### Cell culture and transfection

DU145, PC3, and HEK293 cells were purchased from American Type Culture Collection. DU145 and PC3 cells were cultured in complete RPMI1640 medium supplemented with 2 mM l-glutamine and 10% FBS (Atlanta Biologicals). HEK293 cells were cultured in Dulbecco's modified Eagle's medium with 10% FBS. The transfection was carried out using Lipofectamine 3000 (Thermo Fisher Scientific).

### GST fusion protein pulldown assays

ARF1 activation was measured in GST fusion protein pulldown assays using GST–VHS–GAT fusion proteins in which the GAT domain of GGA3 specifically interacts with the active form of ARF1 as described (19, 52). GST fusion proteins were expressed in bacteria and purified by using MagneGST Glutathione Purification System (Promega) as described previously (53). Purified fusion proteins were analyzed by Coomassie brilliant blue staining following SDS-PAGE before experiments. GST fusion proteins tethered to the glutathione beads were either used immediately or stored at 4 °C for no longer than 2 days.

To measure ARF1 activation by GPCRs, cells were cultured on 12-well dishes for 24 h and starved for 14 h (HEK293 cells) or 48 h (DU145 and PC3 cells). The cells were then stimulated for 5 min with individual GPCR agonists, including UK14304 (1 μM), SDF1α (200 ng/ml), Ang II (1 μM), or adenosine (10 μM). To measure ARF1 activation by inducible Gβγ translocation to the GA, cells were transfected with Gβ1, FRB-Gγ, and Golgi-FKBP (500 ng each) for 24 h and starved as aforementioned before induction with rapamycin at 1 μM for 30 min. After washing with cold PBS twice, the cells were lysed with buffer containing 50 mM Tris–HCl, pH 7.4, 10 mM MgCl_2_, 300 mM NaCl, 2% Nonidet P-40, 0.01% SDS, and 1X Protease Inhibitor Cocktail (Roche). After sonication, total cell lysates were centrifuged at 100,000 rpm for 20 min at 4 °C, and the supernatants were incubated with glutathione beads with gentle rotation at 4 °C overnight. The beads were washed three times with buffer containing 25 mM Tris–HCl, pH 7.4, 30 mM MgCl_2_, 150 mM NaCl, and 1% Nonidet P-40. Active ARF1 bound to the beads was eluted with 2× SDS-gel loading buffer and detected by immunoblotting using ARF1 antibodies.

### Generation of knockout cell lines using the CRISPR–Cas9 genome editing technology

Gγ9, Gγ3, and p110γ knockout cells were generated by using the CRISPR–Cas9 system as described previously (9). Briefly, sgRNAs targeting Gγ9, Gγ3, and p110γ were constructed into the lentiCRISPR v2 vector (Addgene plasmid #52961). The plasmids containing sgRNAs were transfected into cells using Lipofectamine 3000, and the cells were selected in puromycin at a concentration of 10 μg/ml.

### siRNA- and CRISPR–Cas9-mediated depletion of ARF1

siRNA and CRISPR–Cas9 knockout plasmids targeting human ARF1, as well as their controls, were purchased from Santa Cruz Biotechnology. The ARF1 knockout plasmid consists of a pool of three plasmids, each encoding the Cas9 nuclease and a target-specific 20 nt sgRNA. Cells were cultured on 6-well plates and transfected with siRNA (30 nM) or knockout plasmids (1 μg) using Lipofectamine 3000 for 24 h. The cells were then transfected again with the same amount of siRNA or plasmids for another 24 h. To study the effect of ARF1 on ERK1/2 activation by Golgi-Gγ9, the cells were transfected with Gβ1, FRB-Gγ, and Golgi-FKBP (500 ng each) together with siRNA or knockout plasmids in the second transfection. The cells were split at a ratio of 1:2 and grown for additional 24 h and starved before incubation with SDF1α or rapamycin.

### Confocal microscopy

To measure Gβγ translocation, cells were cultured on 25 mm coverslips for 24 h and then transfected with YFP-Gγ9 or YFP-Gγ9-3, together with Gβ1, Gαi1, and pmTurquoise2-Golgi. Before imaging, the cells were starved and then stimulated with SDF1α at 1 μg/ml. The cells were imaged for the YFP and cyan fluorescent protein signals every 5 s using a time-lapse Leica DMi8 microscope (Leica). The translocation of Gβγ to the GA in response to SDF1α stimulation was quantified by measuring the increase of total YFP signal at the GA as described previously (9).

To visualize the subcellular localization of ARF1, cells were cultured on coverslip and starved for 24 h (HEK293 cells) or 48 h (DU145 and PC3). The cells were stimulated with SDF1α at a concentration of 200 ng/ml for 5 min. To study the effect of ARF1 inhibitors on ARF1 localization, cells were treated with secinH3 (100 μM), GCA (30 μM), or Exo2 (60 μM) for 30 min before stimulation with SDF1α. To study the effect of constitutive Gβγ targeting to the GA on ARF1 localization, cells were transiently transfected with Gβ1, FRB-Gγ, and Golgi-FKBP (500 ng each) for 24 h and starved before induction with rapamycin at 1 μM for 30 min. In these experiments, the cells were fixed with 4% paraformaldehyde for 15 min, permeabilized with 0.25% Triton X-100 for 5 min, and blocked with normal donkey serum for 1 h. The cells were then stained with primary antibodies against ARF1 and p230 (1:50 dilution) overnight followed by staining with Alexa Fluor–conjugated secondary antibodies for 1 h. Total ARF1 expression and ARF1 expression at the GA were quantified by the National Institutes of Health ImageJ using p230 as a GA marker. To measure the cell surface expression of CXCR4, cells were cultured on coverslips and transfected with CXCR4–YFP together with siRNA or CRISPR–Cas9 knockout plasmids targeting ARF1. To measure the effect of ARF1 inhibitors on the cell surface expression of CXCR4, cells transfected with CXCR4–YFP were treated with individual inhibitors as described previously. All images were captured using a Zeiss LSM780 confocal microscope as described previously (54).

### Measurement of ERK1/2 activation

ERK1/2 activation in response to stimulation with GPCR agonists and rapamycin-induced Gβγ translocation to the GA was measured as described previously (9, 55, 56). Briefly, cells were grown on 6-well dishes and starved before stimulation with GPCR agonists or rapamycin as indicated in the legends to the figures. The cells were solubilized by the addition of 300 μl of 1× SDS gel-loading buffer, and activation of ERK1/2 was determined by measuring their phosphorylation by immunoblotting.

### Migration and invasion assays

Chemotactic migration of PC3 cells toward SDF1α was quantified using the Boyden migration chambers. Briefly, PC3 cells were suspended in serum-free RPMI1640 medium, and 2 × 10^5^ cells (200 μl) were subjected to transwell migration assays using SDF1α at 200 ng/ml for 48 h at 37 °C. For invasion assays, the suspended cells (2 × 105 cells in 200 μl) were seeded in the top insert coated with diluted Matrigel solution. The migrated and invaded cells were measured by 3-(4,5-dimethylthiazol-2-yl)-2,5-diphenyl-2H-tetrazolium bromide assays and calculated as described previously (9).

### Statistical analysis

Differences were evaluated using Student's *t* test, and *p* < 0.05 was considered as statistically significant. Data are expressed as means ± SD.

## Data availability

All data presented are available upon request from Guangyu Wu (guwu@augusta.edu).

## Supporting information

This article contains supporting information.

## Conflict of interest

The authors declare that they have no conflicts of interest with the contents of this article.
