# Supplementary material for: Gβγ translocation to the Golgi apparatus activates ARF1 to spatiotemporally regulate G protein–coupled receptor signaling to MAPK
Source: J Biol Chem. 2021 May 19;296:100805. doi: 10.1016/j.jbc.2021.100805 (PMC8215300; doi:10.1016/j.jbc.2021.100805)
Supplement: Supplemental Figures S1–S3 [file mmc1.pdf]

Supporting information

Fig. S1

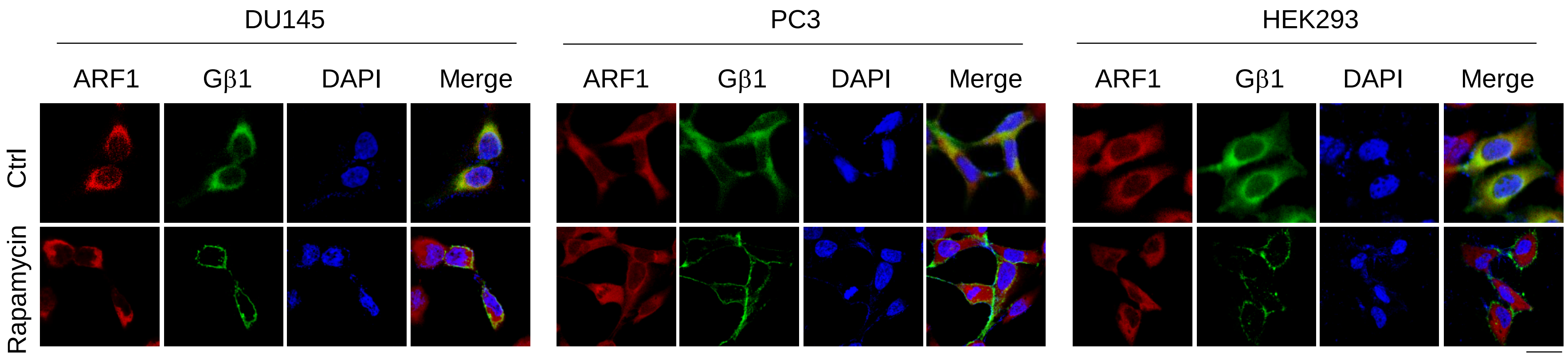

Figure S1. Effect of inducible Gβγ translocation to the PM on the subcellular distribution of ARF1. The cells cultured on coverslip were transfected with FRB-Gγ9, venus-Gβ1 and PM-FKBP, and incubated with rapamycin at 1 μM for 30 min. The images shown are representatives of 2 experiments. Scale bar: 10 μm.

Fig. S2

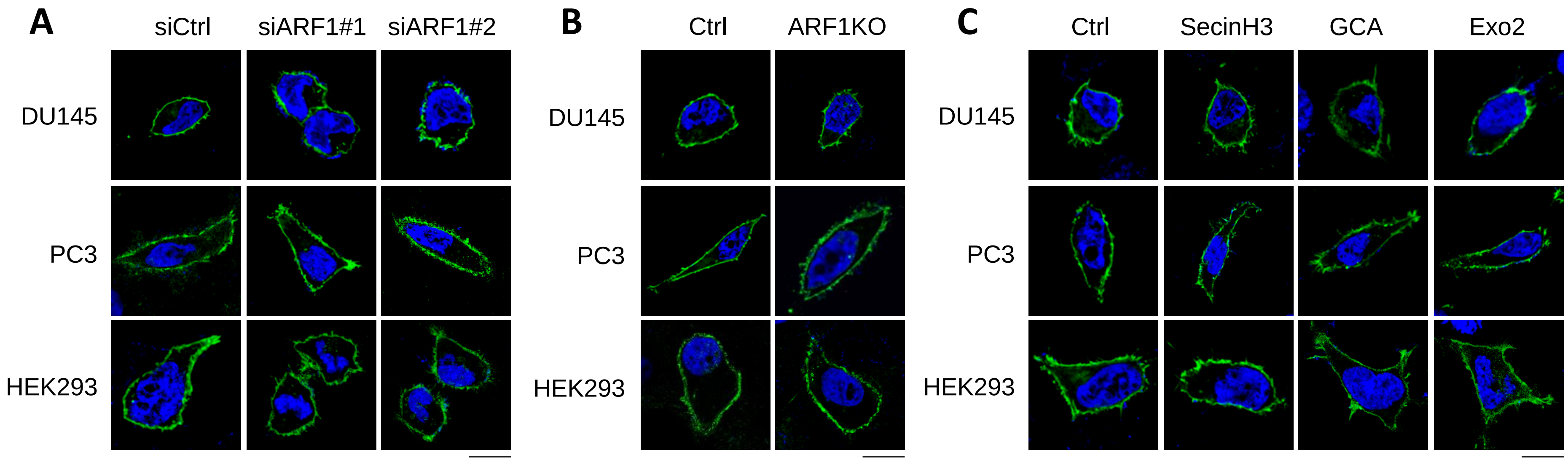

Figure S2. Effects of ARF1 depletion and inhibition on the subcellular localization of CXCR4. A, B, effects of ARF1 depletion by siRNA (A) and CRISPR-Cas9 (B) on the cell surface expression of CXCR. The cells were transfected with siRNA (A) or CRISPR-Cas9 knockout plasmids targeting ARF1 (B), together with CXCR4-YFP. C, effects of ARF1 inhibitors on the cell surface expression of CXCR4. The cells were transfected with CXCR4-YFP and then treated with secinH3 (100 μM), GCA (30 μM) or Exo2 (60 μM) for 30 min. Similar results were obtained in at least three experiments. Scale bars: 10 μm.

Fig. S3

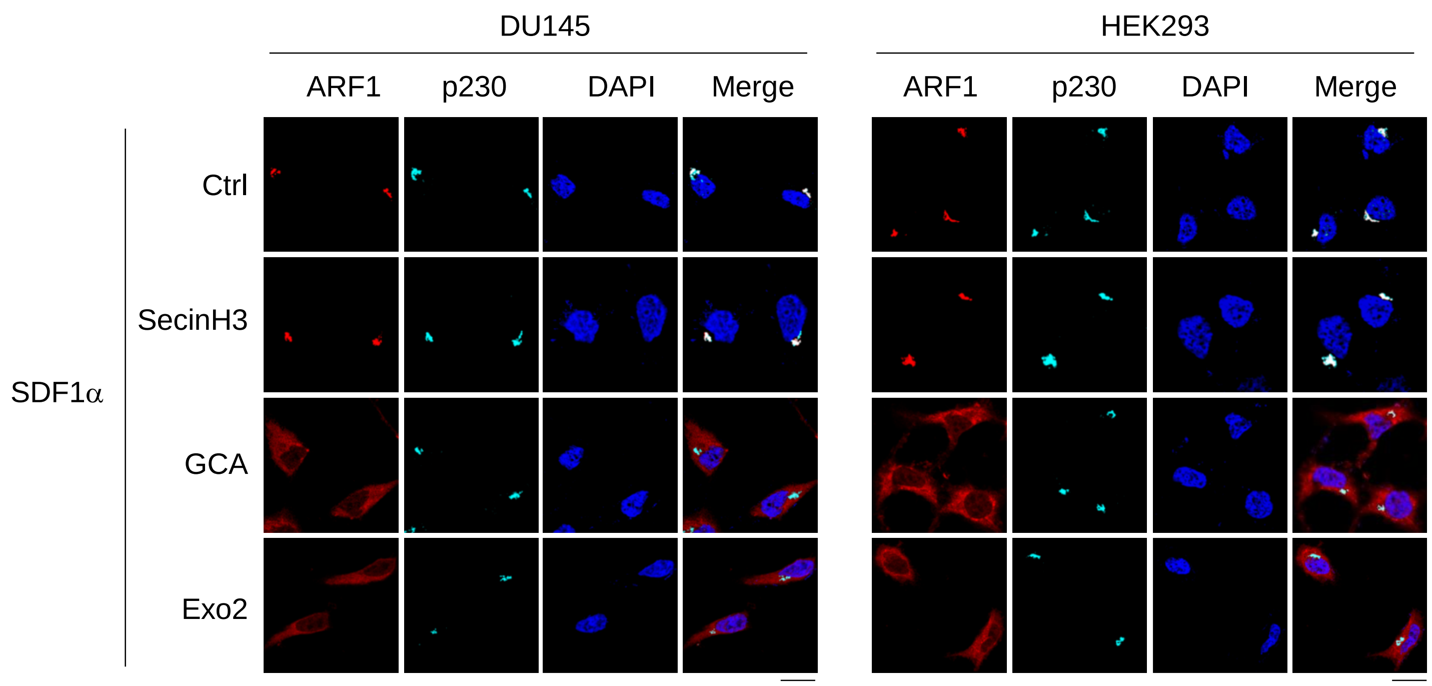

Figure S3. Effects of ARF1 inhibitors on ARF1 localization at the GA. DU145 and HEK293 cells were starved, treated with secinH3 (100 μM), GCA (30 μM) or Exo2 (60 μM) for 30 min, and stimulated with SDF1α at 200 ng/ml for 5 min. The cells were then stained with antibodies against ARF1 and p230. Scale bars: 10 μm.
